# An all-in-one homogeneous DNA walking nanomachine and its application for intracellular analysis of miRNA

**DOI:** 10.7150/thno.36081

**Published:** 2019-08-14

**Authors:** Muren Hu, Dongsheng Mao, Xiaohao Liu, Lingjie Ren, Mengru Zhou, Xiaoxia Chen, Xiaoli Zhu

**Affiliations:** 1 Department of General Surgery, Tongji Hospital, Tongji University School of Medicine, Shanghai 200065, P. R. China; 2 Center for Molecular Recognition and Biosensing, School of Life Sciences, Shanghai University, Shanghai 200444, P. R. China; 3 State Key Laboratory of Oncogenes and Related Genes, Shanghai Cancer Institute, Ren Ji Hospital, School of Medicine, Shanghai Jiao Tong University, Shanghai 200032, P. R. China

**Keywords:** DNA Nanomachine, DNA Walker, MicroRNA, Signal Amplification, Cell Imaging

## Abstract

DNA walker is a powerful type of DNA nanomachine that can produce amplified signals during the "burnt-bridge"-like walking process. Despite their successful application in extracellular bioanalysis, the heterogeneity of the existing DNA walkers makes it difficult to guarantee the consistency of the results during the analysis of different cells.

**Methods**: Here, an all-in-one homogeneous DNA walking nanomachine is reported that can be delivered into living cells for intracellular bioanalysis of miRNA without auxiliary materials.

**Results**: This DNA walking nanomachine is constructed of gold nanoparticles on which two types of interrelated DNA tracks are assembled. The target miRNA, cancer-related miR-21, can be captured by one of the tracks (track 1) and then walk to the other track (track 2), releasing the hybrid of track 1 and track 2 from the nanoparticle to produce a signal. The walking process can proceed in a cyclic 1-2-1-2 manner and thereby produce amplified signals. Thus, sensitive imaging of the miRNA *in situ* can be achieved.

**Conclusion**: Benefiting from the homogeneity of the detection system, the method can be applied for intracellular analysis without interference induced by the fluctuations of stimuli or accessorial contents.

## Introduction

Recently, a growing and exciting field within DNA nanotechnology is the development of DNA nanomachines, which are able to perform mechanical operations on DNA at the nanoscale 1-4. The evolution from simple nanoswitches to integrated nanomachines has brought concept nanodevices 5-8, such as molecular circuits 9, 10, amorphous computations 11, and nanorobots 12, much closer to reality. Several elaborately designed DNA structures have been reported, of which DNA walkers have attracted the most attention 13,14. The mechanical translocation of DNA walkers is usually driven by an external stimulus, including strand exchange reactions, DNAzyme-mediated DNA hydrolysis 15,16, nicking enzymes 17, exonucleases 18, and photoactivated reactions 19,20, and is usually accompanied by DNA tracks on either macroscopic surfaces (e.g., electrodes) 21 or microscopic surfaces (e.g., nanoparticles) 22,23. A "burnt-bridge" event is also associated with the DNA walking motors, resulting in the production of a signal during each step of walking and numerous signals after a certain number of cycles. Therefore, to date, DNA walkers have mainly been applied for the amplified detection of different molecular targets.

Despite their successful application in extracellular bioanalysis, the application of DNA walker-based detection systems for intracellular analysis is hampered by a substantial limitation. In a typical design, a DNA walker-based detection system consists of surface-immobilized DNA tracks, DNA walkers, chemical or physical stimuli, and accessorial reagents 24-26. The transfer of all these materials into cells in a synergetic manner is challenging because the transfer efficiency of each material is different depending on the characteristics of the materials and the cell membrane permeability. To avoid the uncontrollable transfer of multiple components, strategies have been developed by adopting endogenous intracellular substances instead to work as either stimuli or accessorial reagents 27-29. For example, Ye et. al. reported a highly integrated DNA walking nanomachine that could be operated in living cells 30. Endogenous ATP was adopted to work as a stimulus in their design. However, whatever endogenous substances are adopted, their contents vary in different cells and even in different states of the same cell. This uncertainty of the contents is inimical to the precise operation of the DNA walking nanomachine, especially for analysis purposes.

Here, we report an all-in-one DNA walking nanomachine that can be operated in living cells without the need for extra exogenous or endogenous substances. Unlike the conventional fabrication of DNA walkers by combining a swing arm with a single type of DNA tracks, two types of interrelated DNA tracks are assembled on gold nanoparticles in our design. Once one of the tracks (track 1) captures the target miRNA, it undergoes a conformational change and thereafter migrates together with the target to the other type of track (track 2). Subsequently, the miRNA walks further to a new track 1, leaving the hybrid of pre-passed track 1 and track 2 free from the nanoparticles. Thus, the target miRNA takes turns walking between the two types of tracks and thereby produces amplified signals without the aid of any other stimuli or accessorial reagents. In this way, we can analyze intracellular targets without interference induced by the fluctuations of stimulus contents. Furthermore, in comparison with certain reported methods for miRNA imaging in which either tool enzymes (e.g., polymerase) or additional carriers (e.g., liposome) are required, our strategy is composed of a simple system integrating the sensing element and the carrier and thus has the advantage of simplicity.

## Materials and Methods

All chemicals were of analytical grade and were obtained from Sigma-Aldrich Inc. (St. Louis, MO). Phosphate-buffered saline (PBS, 20×), trypsin, 3-(4,5-dimethylthiazol-2-yl)-2-diphenyltetrazolium bromide (MTT), and cell culture media (RPMI-1640/DMEM) were obtained from KeyGen Biotech Co., Ltd. (Nanjing, China). All cell lines (HeLa, MCF-7 and L02) were from the Cell Bank of the Committee on Type Culture Collection of the Chinese Academy of Sciences (Shanghai, China). Water used in this work was RNase free. Syringe-driven filters (0.22 μm), all oligonucleotides (HPLC purified, sequences shown in Table S1), DNase I and the electrophoresis reagents used in this work were obtained from Sangon Biotechnology Co., Ltd. (Shanghai, China).

**Fluorescence measurements***.* FITC fluorescence spectra were measured between 500 nm and 540 nm using a maximal excitation wavelength of 494 nm. The optimum excitation and emission wavelengths for TAMRA were determined using a similar method, and the peak fluorescence emission was recorded at an excitation wavelength of 558 nm. Two groups of HP-1 mixed with HP-2 at a 1:1 molar ratio were evaluated. The fluorescence intensity in the first group was recorded immediately using a maximal excitation wavelength of 494 nm. The second group was incubated at 95 ℃ for 5 min and then gradually cooled to room temperature for fully complementary matching. Then, the FRET signal was measured at emission wavelengths between 500 nm and 620 nm after excitation at 494 nm. All the fluorescence measurements were carried out using an F-7000 fluorescence spectrophotometer (Hitachi, Japan).

**Synthesis of nanomachine**. The synthesis of the nanomachine was based on thiolated gold nanoparticles (AuNPs). First, the gold nanoparticles were synthesized using the classical sodium citrate reduction method. Thiol-modified loading sequences (LS-1/LS-2) were separately reduced by 10 mM TCEP at 37 ℃ for 1 h. Then, the hairpin 1 (HP-1) and loading sequence 1 (LS-1) constructs were mixed at a 1:1 proportion at 95 ℃ for 5 min and gradually cooled to room temperature before use. The procedure for mixing HP-2 with LS-2 (1:1) to facilitate complete hybridization was the same as that described previously. The two mixed solutions (HP-1/LS-1, HP-2/LS-2) were added to AuNP solutions and then shaken gently overnight at 37 °C. Subsequently, a salt aging process was conducted by adding 2 M sodium chloride solution, causing more DNA molecules to bind to the nanoparticles. Finally, the nanomachine was centrifuged at 12,000 rpm for 30 min and washed three times with 0.01 M PBS. The nanoparticles were dispersed in PBS and stored at 4 ℃. The sizes of the AuNPs and nanomachine were measured with a ZETASIZER 3000HS instrument (Malvern Instruments Ltd., UK). UV-vis spectra of the AuNPs and nanomachine were recorded with a UV-2450 spectrophotometer (Shimadzu, Japan). The AuNP concentration was calculated based on the Beer-Lambert law.

**Optimization of toehold length**. HP-1 oligonucleotides with different toehold lengths (4 nt, 6 nt and 8 nt) were synthesized by Sangon Biotechnology Co., Ltd. The constructed nanomachines with different toehold lengths (4 nt, 6 nt and 8 nt) were treated with 200 nM miR-21 in 1×PBS. After incubation at 37 ℃ for 3 h, the real time FRET signals in the supernatant were measured after centrifugation.

**Quantification of hairpin loading on each AuNP**. First, a fluorescence standard curve generated using FITC-labeled HP-1 at different concentrations was constructed. Then, HP-1 mixed with LS-1 was loaded onto AuNPs for quantification. The mixture was washed with PBS three times to remove unbound HP-1 and LS-1. The sediment was treated with a 10 mM solution of DTT to completely release HP-1 from the AuNPs. After centrifugation, the supernatant was collected for fluorimetric determination. The fluorescence of FITC on HP-1 was excited at 494 nm and measured at 517 nm. The number of hairpins on each AuNP was determined based on a comparison to the standard curve.

To quantify the ratio of HP-1 to HP-2 on AuNPs, we treated the nanomachine that was functionalized with equal amounts of FITC-labeled HP-1 and TAMRA-labeled HP-2 by using DTT (10 mM) for 1 h to dissociate all the HP-1 and HP-2 from the AuNPs, respectively. The fluorescences of HP-1 and HP-2 in the supernatant after centrifugation were detected and referred to their respective standard curves for quantification. The fluorescence of FITC on HP-1 was excited at 494 nm and measured at 517 nm. The fluorescence of TAMRA on HP-2 was excited at 558 nm and measured at 583 nm.

**Stability of nanomachines***.* Freshly prepared nanomachines were treated with PBS, DMEM with 10% FBS, RPMI-1640 with 10% FBS, DNase I, cell lysate, or miR-21 for 3 h. The fluorescence intensities of the hairpins were measured at a 494 nm excitation wavelength.

**Detection of miRNAs *in vitro*.** For specific *in vitro* detection of miR-21, nanomachines were incubated with different miRNAs (miR-16, miR-26a, miR-214, and miR-21). After incubation for 3 h at 37 ℃, the fluorescence of the supernatant after centrifugation was measured. Different concentrations of the miR-21 target were added to the nanomachine solution. After reaction at 37 °C for 3 h, the mixture was centrifuged to obtain the supernatant for further electrophoresis.

**Cell culture.** L02 cells (human normal liver cells) and MCF-7 cells (human breast cancer cells) were cultured in RPMI-1640 medium (Gibco) supplemented with 10% fetal bovine serum (FBS), penicillin (100 units mL^-1^), and streptomycin (100 μg mL^-1^) in a humidified atmosphere containing 5% CO_2_ at 37 °C. HeLa cells (human cervical cancer cells) were cultured in DMEM supplemented with 10% FBS, penicillin (100 units mL^-1^), and streptomycin (100 μg mL^-1^). All cells were collected during the exponential phase of growth for further use.

**Confocal fluorescence imaging.** MCF-7 cells, HeLa cells and L02 cells were added to 35 mm^2^ confocal dishes in culture medium at 37 °C and incubated for 24 h to reach 80% confluency. Then, the cells were washed with PBS three times. Sterile filtered nanomachines (2 nM and 6 nM) were added to the confocal dishes in RPMI-1640 or DMEM cell culture medium and incubated with cells for 3 h. Then, the cells were washed with PBS three times to remove nanoparticles attached to the surface of cells. Nuclei were stained with DAPI, and cells were washed with 1×PBS thrice. Fluorescence images of the cells were acquired under a confocal laser scanning microscope at an excitation wavelength of 405 nm and 488 nm.

For imaging of cells with miRNA mimics and inhibitors, a standard protocol was followed. With Hieff Trans Liposomal transfection reagent, 20 pM miR-21 mimics and inhibitors were transfected into HeLa cells for 48 h. The cells were then treated with nanomachine (6 nM) for 3 h as described above. The cells were imaged with a confocal laser scanning microscope.

**Intracellular co-localization of lysosomes and the nanomachine.** MCF-7 cells were planted in confocal dishes for 24 h. Subsequently, the cells were incubated with the nanomachine (2 nM) for 1 h or 3 h. Then, LysoTracker blue (1 μM) was added to the cells for co-incubation for 30 min, after which the cells were observed with a confocal microscope.

**Detection of microRNAs via qRT-PCR.** Total RNA was extracted from MCF-7, HeLa and L02 cells using TRIzol reagent according to the standard RNA extraction protocol. cDNA was obtained by reverse transcription using the iScriptTM cDNA synthesis kit (Bio-Rad, USA). qRT-PCR analysis of miRNA was performed with an ABI 7900HT FAST Real-Time PCR system (Applied Biosystems, Foster City, USA). The U6 was used as the internal control for miR-21, and the 2^-ΔΔCt^ method was used to calculate the relative expression of miR-21. The primers used in this experiment are described in Table S1.

**Imaging of microRNAs *via* fluorescence *in situ* hybridization (FISH).** MCF-7, HeLa and L02 cells were seeded in confocal dishes for 24 h at 37 ℃ and then fixed by 4% paraformaldehyde. The confocal dishes were immersed in proteinase k buffer at 37 ℃ for 20 min and then washed with RNA-free water. The sample was dehydrated with ethanol gradients and then dried. Hybrid droplets containing molecular beacons were added to the sample and hybridized in a wet box containing 2×SSC for 16 h in the dark. The confocal dishes were washed three times and then observed with a confocal microscope.

**Determination of cell viability.** MTT assays were carried out to evaluate the potential cytotoxicity of nanomachines toward different cell lines. MCF-7 cells (1 × 10^4^ cells mL^-1^) were seeded in 96-well plates in RPMI-1640 culture medium in a humidified atmosphere containing 5% CO_2_ at 37 °C. Subsequently, nanomachines (6 nM) were added to each well for different time intervals (0, 3, 6, 12, 18, or 24 h). Cells incubated with only medium served as the controls. After removal of the medium, the cells were cultured with FBS-free medium for 48 h and treated with 50 μL of 1×MTT solution at 37 ℃ for 4 h to form formazan. Then, 150 μL of DMSO was added to completely dissolve the formazan crystals. The absorbance was measured at 550 nm on a microplate reader to calculate cell viability.

## Results and Discussion

**Scheme 1:**
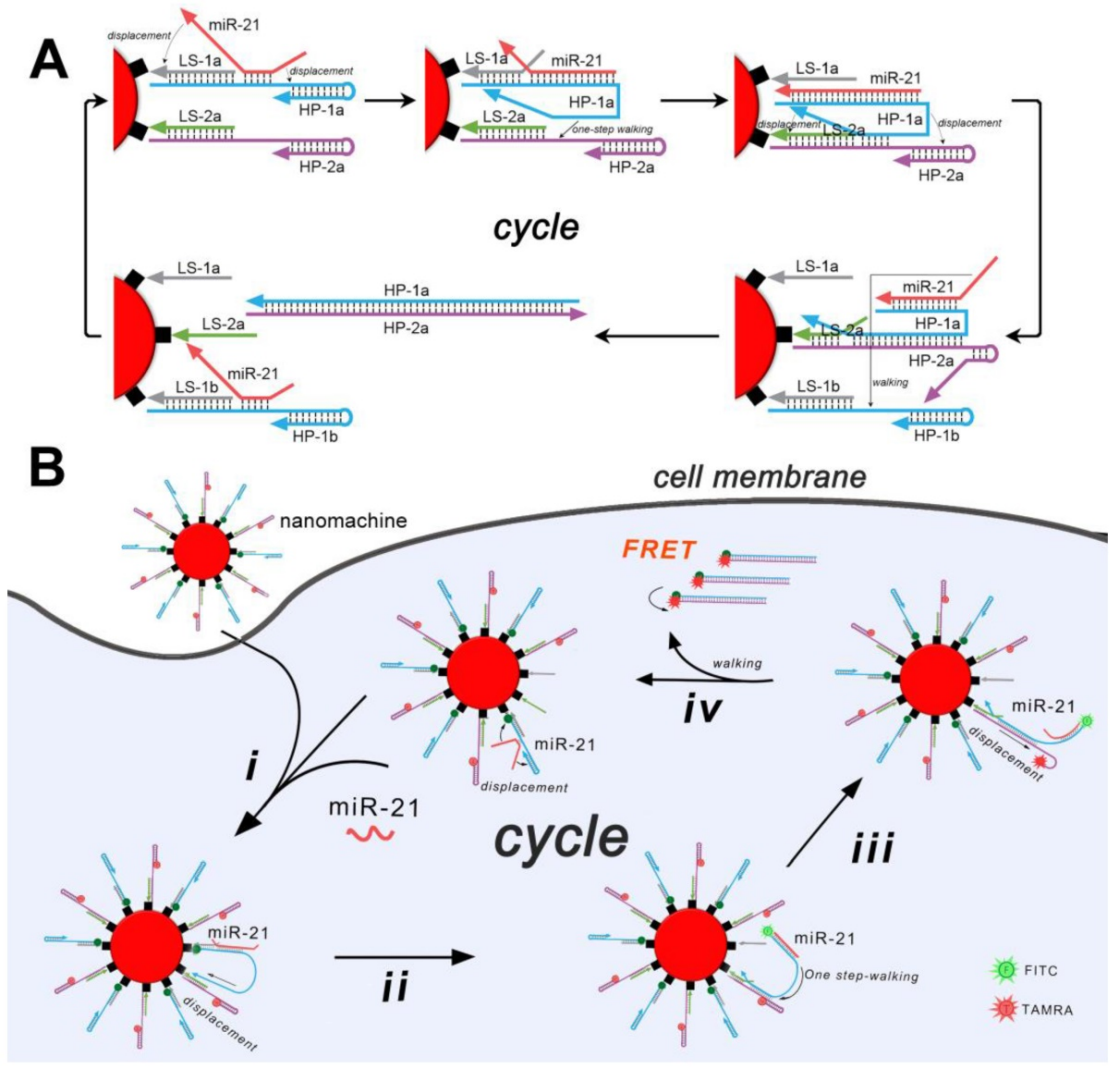
Schematic presentation of the all-in-one DNA walking nanomachine. (A) Scheme of the design of the DNA walking nanomachine showing how it walks on a nanoparticle surface. (B) Scheme of the DNA walking nanomachine for miRNA analysis in living cells.

As is shown in Scheme 1A, gold nanoparticles are first functionalized with two types of loading DNA strands (LS-1 and -2), which are able to recruit corresponding two types of hairpins (HP-1 and -2) through base pairing. The loading strands and the hairpins work together as DNA tracks, in which the loading strands are immobilized tracks and the hairpins are mobilizable tracks. This well-designed architecture is able to capture the walker (miR-21, also working as the target) through an exposed toehold region on HP-1. Once captured, the walker undergoes strand displacements automatically at both terminals. The strand displacement at the 3' terminal will result in the release of the HP-1 from the LS-1, and the displacement at the 5' terminal will unfold the internal stem of HP-1. The HP-1 with conformational changes will further migrate to a proximate HP-2 through base pairing with the toehold region on HP-2. Similarly, strand displacements occur at both terminals of HP-1. In this case, the strand displacement at the 3' terminal of HP-1 results in the release of the HP-2 from the LS-2, and the displacement at the 5' terminal unfolds the internal stem of HP-2. As a result, a hybrid of HP-1 and HP-2 is produced and is released from the nanoparticles. The displaced miRNA migrates further to a proximate new HP-1 and thereafter launches a new round of "burnt-bridge" walking. Overall, the target walks on the two types of DNA tracks, releasing the mobilizable tracks (HP-1 and -2) from the immobilized tracks (LS-1 and -2) as a result. Each step of the walking produces one molecule of the hybrid of HP-1 and -2, and an accumulation of the hybrid will be produced after continuous walking on the nanoparticles. To achieve the biosensing of the target, the HP-1 and -2 are labeled with fluorophore FITC and TAMRA, respectively. While being functionalized on the surface of the gold nanoparticles, their fluorescence is quenched by the nanoparticles. After the target-induced walking, the flares of the produced free HP-1 and -2 are recovered (Scheme 1B). Furthermore, to eliminate unspecific false-positive fluorescence signals, which are common when using a single fluorescence probe or molecular beacon and usually originate from the hydrolysis of endogenous nucleases, fluorescence resonance energy transfer (FRET) between the donor FITC and acceptor TAMRA is monitored. Only in the case that the undegraded HP-1 and -2 hybridize and bring the donor and acceptor in close proximity can a FRET signal be obtained (Figure S1).

**Figure 1:**
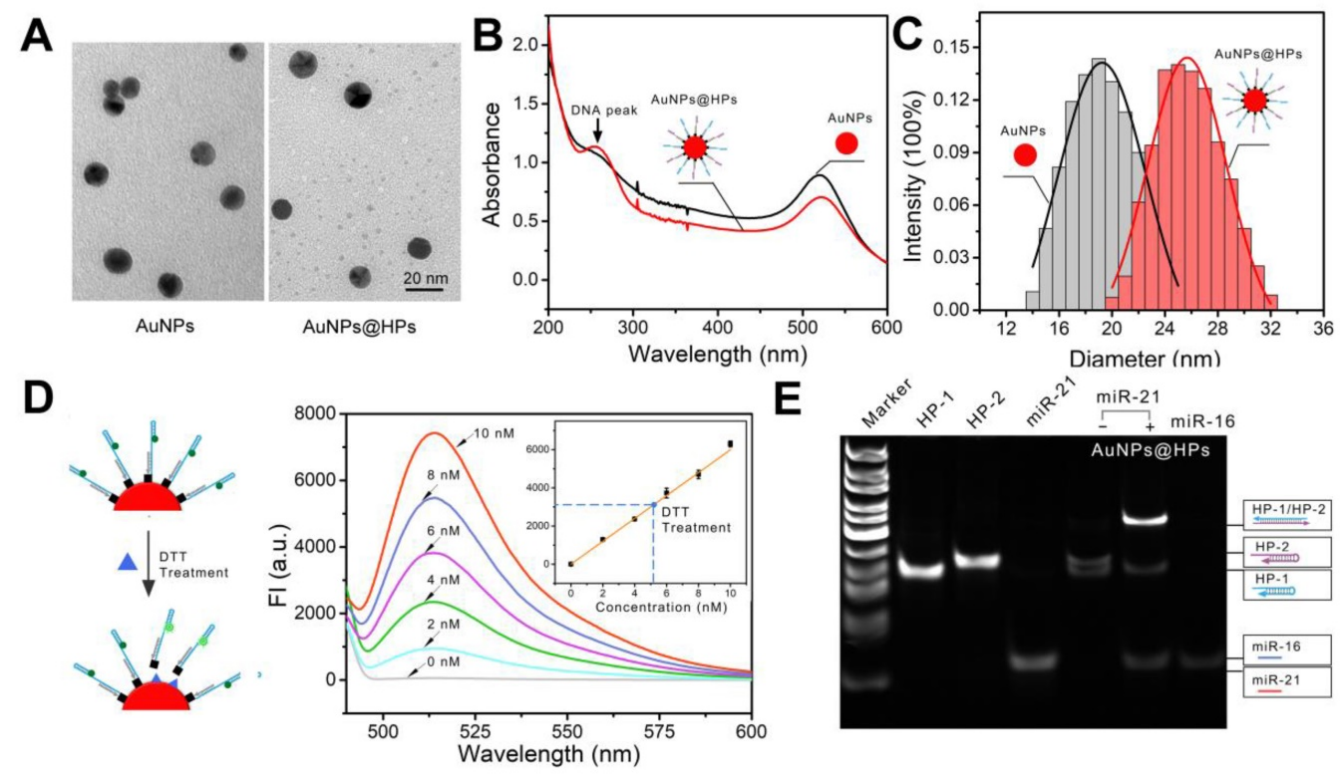
Characterization of the nanomachine. (A) TEM images of AuNPs and AuNPs@HPs. (B) UV-vis spectra of naked AuNPs and functionalized AuNPs. (C) Dynamic light scattering (DLS) of naked AuNPs and functionalized AuNPs. (D) Quantitative analysis of HPs on AuNPs. Left: Scheme of the functionalized AuNPs after treatment with DTT. Right: Fluorescence spectra of HP-1 at different concentrations. Insert: Standard linear calibration curve of the fluorescence intensity against the concentration of HP-1. The dash line intersection represents the fluorescence intensity of HP-1 dissociated from AuNPs after DTT treatment and the corresponding concentration. (E) Electrophoretic patterns showing the DNA ladder (lane 1), HP-1 (lane 2), HP-2 (lane 3), miR-21 (lane 4), products dissociated from the nanomachine triggered by nothing (lane 5) or the target miR-21 (lane 6).

The all-in-one DNA walking nanomachine was manufactured by functionalizing gold nanoparticles (AuNPs) with thiol-tagged oligonucleotides (LS-1/HP-1, LS-2/HP-2). TEM images intuitively demonstrated the morphology and good dispersity of the AuNPs and functionalized AuNPs (Figure 1A). The UV-vis absorption spectra showed that the characteristic peak of the AuNPs was redshifted from 519 nm to 521 nm after being functionalized. An obvious characteristic DNA peak at 260 nm was also present (Figure 1B), confirming conjugation of the oligonucleotides to the surface of AuNPs. Additionally, due to the conjugation of oligonucleotides, the diameter of the functionalized nanoparticles increased to 25 nm compared to the 19 nm bare gold nanoparticles (Figure 1C). Furthermore, the examination of thiolated gold nanoconjugates in the presence of dithiothreitol (DTT) is considered to work as a standard test in quantitative characterization of the conjugated oligonucleotides. The average number of HPs loaded onto each gold nanoparticle was calculated to be approximately 101 based on the standard curve of the fluorescence quantitative analysis of the fluorophore-labeled oligonucleotides (Figure 1D). The ratio of HP-1 to HP-2 was also confirmed to be approximately 1:1 by quantifying each of them on the AuNPs (Figure S2). Overall, the above experimental results suggested the structural completion of the all-in-one DNA walking nanomachine.

To confirm the general prediction that the target (miR-21) propelled "burnt-bridge" walking on the surface of AuNPs, a gel electrophoresis experiment was conducted to characterize the release of the HPs from the AuNPs (Figure 1E). The assembled nanoparticles were incubated with the target miR-21 at 37 °C for 3 h. Subsequently, the supernatant solution of the nanoconjugates was obtained for gel electrophoresis. A bright band with the high-molecular-weight characteristic of the hybrid of HP-1 and HP-2 was observed (Figure 1E, lane 6), suggesting the successful proceeding of the walker and the release of the products. In the absence of the target (lane 5), the walking could not be activated and no HP-1/HP-2 hybrid could be observed, suggesting that only in the presence of the trigger miR-21 can nanomachines provide power to start working.

**Figure 2:**
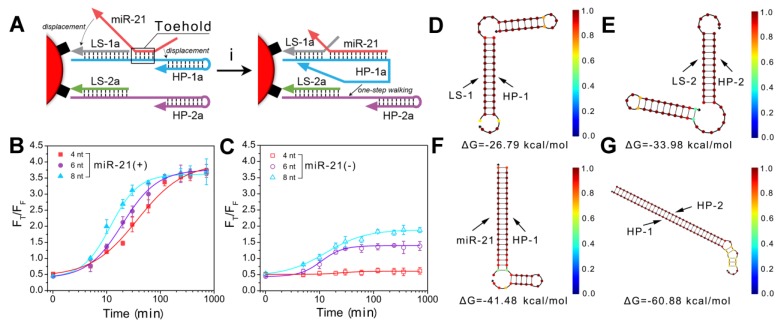
Characterization of the behavior of toehold-mediated "burnt-bridge" walking. (A) Scheme showing the toehold-mediated "burnt-bridge" walking on the surface of nanoparticles. (B, C) FRET ratio (F_T_/F_F_) *vs.* time showing the reaction kinetics of different toehold lengths of the nanomachine in the presence (B) or absence (C) of miR-21. (D-G) The free energy and structure of HP-1/LS-1 (D), HP-2/LS-2 (E), HP-1/miR-21 complex (F) and HP-1/HP-2 complex (G). The data were obtained by NUPACK.

Because the essential component of the all-in-one DNA walking nanomachine is the toehold-mediated strand displacement, it is necessary to characterize the behavior of nanomachines bearing different lengths of the toehold domain. First, we studied the reaction kinetics of different toehold lengths (4, 6 and 8 bases) (Figure 2A, B). The results indicated that the longer the toehold was, the more likely the strand displacement was to be triggered by the target. However, the two legs of HP-1 and HP-2 with longer toehold lengths were also more likely to hybridize in the absence of targets, producing higher background signals (Figure 2C). Considering the signal to noise ratio, 4 bases was selected as the optimum toehold length. In addition, we monitored the dynamics of the nanomachine. The results showed that the nanomachine could be launched in 5 min and completed within 2 h (Figure 2B), suggesting a rapid response. Furthermore, the free energy of HP complexation was investigated using NUPACK, an online software system for analysis and design of nucleic acid structures. In the absence of target miRNA (miR-21), the hairpins (HP-1 and HP-2) were trapped in a metastable state with -26.79 kcal/mol and -33.98 kcal/mol free energies, respectively, and incapable of spontaneous hybridization with each other (Figure 2D, E). Once in the presence of the initiator miR-21, HP-1 captured the target of interest with -41.48 kcal/mol free energy (Figure 2F). Subsequently, strand displacements occurred with significantly reduced free energy from -41.48 kcal/mol (miR-21/HP-1) to -60.88 kcal/mol (HP-1/HP-2), which indicated that conditions favored the formation of double-stranded DNA helices (HP-1/HP-2) (Figure 2G).

**Figure 3:**
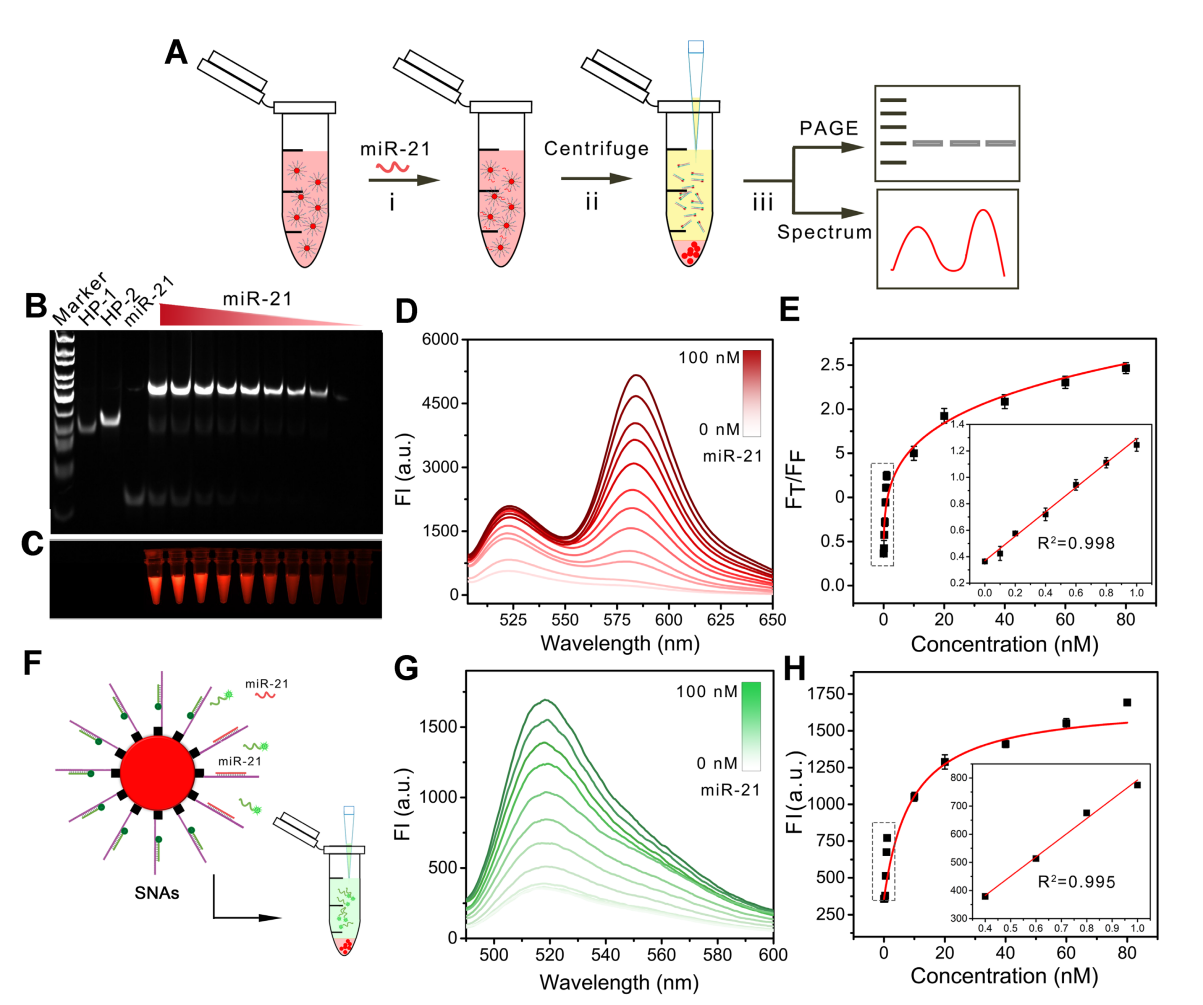
The operation of nanomachine for miRNA analysis *in vitro*. (A) Scheme showing the procedure for gel electrophoretic analysis and fluorescence determination of the products from the walking reaction. (B) Gel electrophoretic analysis of the products of the walking reaction triggered by miR-21 with different concentrations. (C) Fluorescent image of walking products. (D) Fluorescence spectra of released HP-1/HP-2 in the presence of different concentrations of miR-21 at an excitation wavelength of 494 nm. (E) Calibration curve of F_T_/F_F_ *vs.* the concentrations of miR-21. Inset: linear relationship between the fluorescence ratio and the concentrations of miR-21. (F) Scheme of spherical nucleic acids (SNAs) for the analysis of miRNA. (G) Fluorescence spectra of SNAs recorded in the presence of miR-21 at different concentrations. (H) Calibration curve of fluorescence intensity *vs.* the concentrations of miR-21. Inset: linear relationship between the fluorescence intensities and the concentrations of miR-21.

After the successful construction of the nanomachine, we further explored its performance *in vitro*. We evaluated the operation of the DNA walking nanomachine under different concentrations of triggers,* i.e.,* the target miR-21. The hybridization products after walking were centrifuged for TBE-PAGE electrophoresis and fluorescence determination (Figure 3A). The results of electrophoresis demonstrated that the turnover traveled along the surface of the nanoparticles with two types of interrelated DNA tracks and induced hybridization. Furthermore, the walking products increased as the concentration of target increased (Figure 3B, C). The fluorescence results of FRET displayed a similar tendency in which the values of F_T_/F_F_ increased linearly as the concentration of the target miR-21 increased from 26 pM to 1 nM. The limit of detection (LOD) was calculated to be 26 pM (Figure 3D, E). The detection sensitivity of the nanomachine was significantly improved in comparison with traditional spherical nucleic acids (SNAs), whose detection limit was calculated to be 1.3 nM (Figure 3F-H). Table S2 also shows that the assay performance of the nanomachine was comparable to or better than some previously reported methods for intracellular miRNA detection. The results demonstrate that the DNA walking nanomachine possesses outstanding signal amplification ability and can potentially be used for highly sensitive bioanalysis.

To use this nanomachine for intracellular bioanalysis, it is essential to explore its stability. A nuclease, DNase I endonuclease, that plays an important role in biodegrading dsDNA and ssDNA in living cells was added to the solution of the nanomachine at 37 ℃ for 3 h. After centrifugation, the supernatant was collected for fluorescence spectrometry analysis. As shown in Figure S3, the FRET signals in the presence of the nuclease could not be generated, suggesting that no fluorophore-labeled oligonucleotides were released and that the nanomachine was stable. In addition, the stability of the nanomachine was studied in different media, namely, PBS, DMEM with 10% FBS, and RPMI-1640 with 10% FBS. No significant signals could be observed. In contrast, the relative fluorescence intensity (F_T_/F_F_) exhibited a significant increase upon addition of miR-21 or miRNA-containing cell lysate. Collectively, the above results verify that the nanomachine has good stability, which provides the basis for cellular and *in vivo* applications.

To further investigate the specificity of the nanomachine, we tested the response of the nanomachine to different miRNAs. Those miRNAs with a similar sequence to miR-21 were picked from an miRNA database and were adopted as controls. As shown in Figure S4, the nanomachine was unresponsive to these nonhomologous miRNAs, demonstrating that the proposed DNA walking nanomachine has good specificity for target detection.

Upon fabrication of the DNA walking nanomachine *in vitro*, we studied its analytical performance for intracellular analysis. First, the cytotoxicity of the nanomachine was assessed by MTT assays. The results showed that cell viability was maintained at 88% after incubation with the nanomachine for 24 h, suggesting a low cytotoxicity and excellent biocompatibility of the nanomachine (Figure S5). Next, to obtain the optimum intracellular analysis, we optimized the incubation time of the nanomachine with cells. Confocal images were taken after incubation of the nanomachine with MCF-7 cells at different time points. As shown in Figure S6, FRET signals of bright red fluorescence increased in MCF-7 cells from 1 h to 3 h and then decreased. Thus, 3 h was selected as the optimal incubation time for subsequent experiments. Co-localization of the nanomachine with lysosomes was further conducted to determine whether the nanomachine could escape from the lysosomes, which is a vital prerequisite for intracellular analysis of miRNAs. Figure S7 shows that the nanomachine achieved effective lysosomal escape after 3 h incubation with the cells.

**Figure 4:**
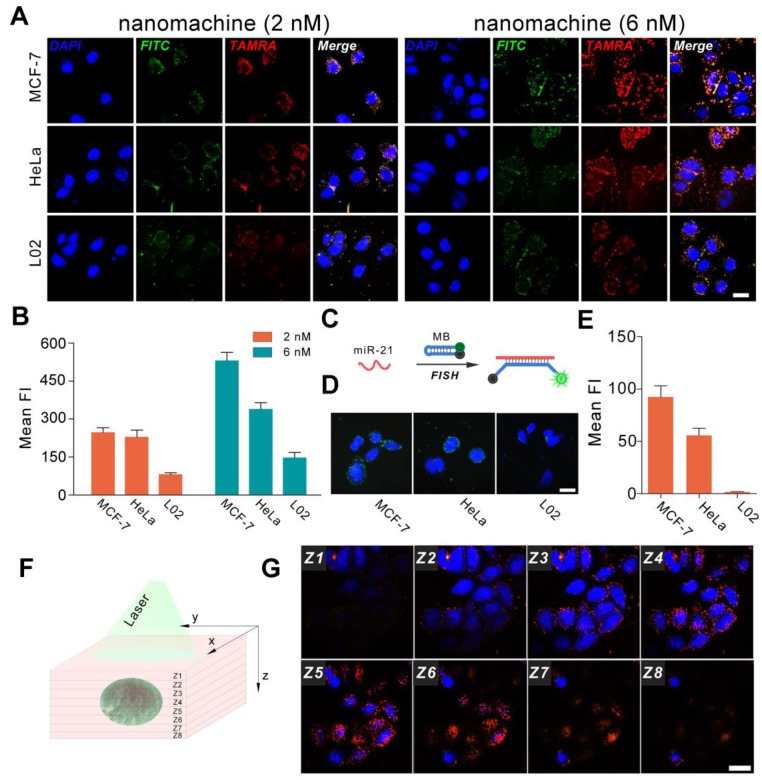
Confocal images of the nanomachine for intracellular analysis of miRNA. (A) Confocal images of MCF-7, HeLa and L02 cells incubated with nanomachines of different concentrations (2 nM and 6 nM). (B) Histogram of fluorescence intensity of (A) measured by Image J 1.51. (C) Scheme of fluorescence *in situ* hybridization. (D) Fluorescence *in situ* hybridization (FISH) of miR-21 in MCF-7, HeLa and L02 cells. (E) Histogram of fluorescence intensity of (D) measured by Image J 1.51. (F) Scheme of Z-stack images acquisition. (G) Z-stack images of MCF-7 cells incubated with nanomachine (6 nM) for 3 h. Both the red and green channels were excited at 488 nm. Scale bar: 20 μm.

To evaluate the universality of the DNA walking nanomachine, three cell lines (MCF-7, HeLa and L02) with varying miR-21 expression levels were chosen as model cells. The results of qRT-PCR showed that there was a gradient of the miR-21 expression: MCF-7 > HeLa > L02 (Figure S8). Thus, MCF-7 was considered positive for cell lines with higher miR-21 expression, and L02 cells represented the control cell line with lower miR-21 expression. Nanomachines with two different concentrations (2 nM and 6 nM) were incubated with the cells for 3 h. When the 2 nM nanomachine was employed, the FRET intensity in MCF-7 and HeLa cells was much higher than that in L02 cells, representing an expected bright red fluorescence (Figure 4A, B). However, the FRET intensity in MCF-7 cells was comparable to that in HeLa cells, possibly because the FRET signals were saturated by incubation with a low dose of nanomachine in living cells, causing all the hairpins to dissociate from the gold nanoparticles. In the presence of a relatively high concentration of the nanomachine (6 nM), the problem was resolved. Successful differentiation of the miRNA expression profiles among the three cell lines was achieved. In this case, the amount of the nanomachine is sufficient to present discriminable signals. In comparison with fluorescence *in situ* hybridization (FISH), a conventional method for the study of miRNA *in situ*, the fluorescence intensity of our method increased by approximately 6, 6 and 140-fold for MCF-7, HeLa and L02 cells, respectively, suggesting a good sensitivity (Figure 4C-E). Specifically, the minimally abundant miRNA in L02 cells was almost unobservable when using FISH but presents bright fluorescence when using our DNA walking nanomachine. Further analysis of Z-stack images revealed that the cell fluorescence gradually increased and then disappeared from the bottom to the top of the cells, indicating that the signals of nanomachine originated from the cytoplasm, where the target miRNA was located (Figure 4F, G). Monitoring the regulation of the target miRNA was also achieved. The imaging results show that both up-regulation by a miR-21 mimic and down-regulation by a miR-21 inhibitor can be well discriminated using our DNA nanomachine (Figure S9).

## Conclusion

In conclusion, we demonstrated an all-in-one DNA walking nanomachine powered by endogenous miRNA to execute two tracks walking on the surface of nanoparticles. Unlike other nanomachines that require exogenous components and fuel as a power source, which severely hindered access to the interacting components and efficient operation of machines, this method eliminates the need for exogenous delivery, making the walking process more autonomous and productive in complex cellular environments. In addition, the FRET signals generated by the walking of the two tracks are able to effectively eliminate false-positive results that are common in some reports using only one fluorophore. Further, our designed nanomachine can achieve 1-N times signal amplification in living cells based on a "burnt-bridge" walking, providing a sensitive analysis of intracellular miRNA. By further introduction aptamer or antibody-DNA conjugation to launch the DNA walking nanomachine, the nanomachine presented here also has the potential to be applied for intracellular analysis of other types of targets, e.g., small molecules and proteins. We believe that this work will provide a useful reference for addressing the issues of exogenous delivery and unsatisfactory sensitivity analysis of current nanomachines.

## Supplementary Material

Supplementary figures and tables.

## Abbreviations

FRET: fluorescence resonance energy transfer
AuNPs: gold nanoparticles
DTT: dithiothreitol
LOD: limit of detection
SNAs: spherical nucleic acids
FISH: fluorescence *in situ* hybridization
