# Supplementary material for: An all-in-one homogeneous DNA walking nanomachine and its application for intracellular analysis of miRNA
Source: Theranostics. 2019 Aug 14;9(20):5914–23. doi: 10.7150/thno.36081 (PMC6735375; doi:10.7150/thno.36081)
Supplement: Supplementary file 1 — Supplementary figures and tables. [file thnov09p5914s1.pdf]

An all-in-one homogeneous DNA walking nanomachine and its application for intracellular analysis of miRNA

Muren Hu[1], Dongsheng Mao[2], Xiaohao Liu[2], Lingjie Ren[2], Mengru Zhou[2], Xiaoxia Chen[2,3,*], and Xiaoli Zhu[2,*]

[1] Department of General Surgery, Tongji Hospital, Tongji University School of Medicine, Shanghai 200065, P. R. China

[2]Center for Molecular Recognition and Biosensing, School of Life Sciences, Shanghai University, Shanghai 200444, P. R. China

[3]State Key Laboratory of Oncogenes and Related Genes, Shanghai Cancer Institute, Ren Ji Hospital, School of Medicine, Shanghai Jiao Tong University, Shanghai 200032, P. R. China

[*]Corresponding authors: accalia@sjtu.edu.cn; xiaolizhu@shu.edu.cn

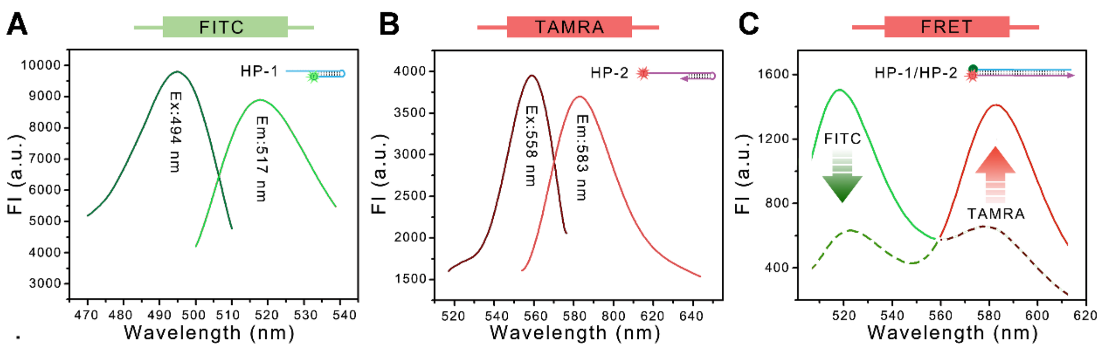

Figure S1 FRET induced by the hybridization of HP-1 (green) and HP-2 (red). (A) Fluorescence excitation and emission spectra of FITC. (B) Fluorescence excitation and emission spectra of TAMRA. (C) The occurrence of FRET of HP-1/HP-2 after annealing.

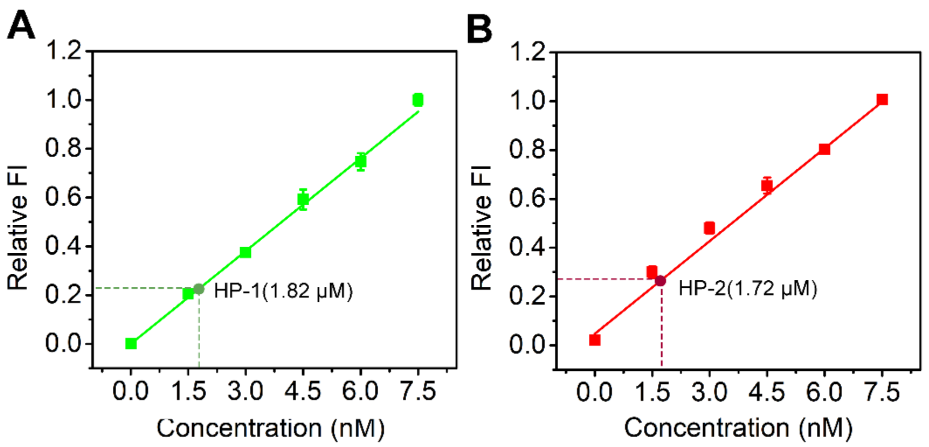

Figure S2 Standard linear calibration curves of (A) FITC-labeled HP-1 (Ex: 494 nm; Em: 517 nm) and (B) TAMRA-labeled HP-2 (Ex: 558 nm; Em: 583 nm). The intersection points of the dashed lines show the relative fluorescence intensities of HP-1 and HP-2 released from the AuNPs using DTT (10 mM) treatment.

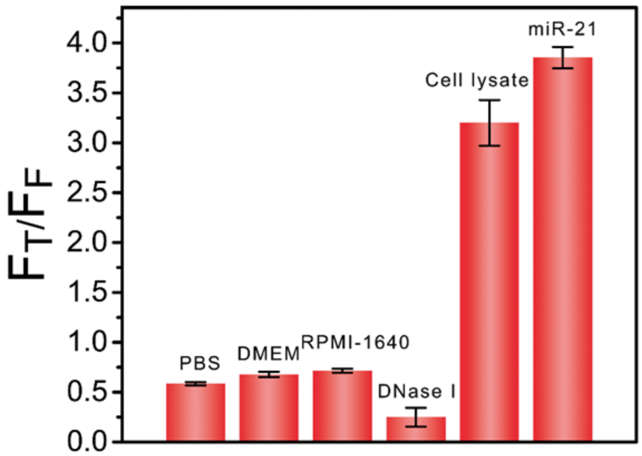

Figure S3 Fluorescence intensity ratio (F_T/F_F) of the nanomachine in the presence of PBS, DMEM with 10% FBS, RPMI-1640 with 10% FBS, DNase I, cell lysate and miR-21.

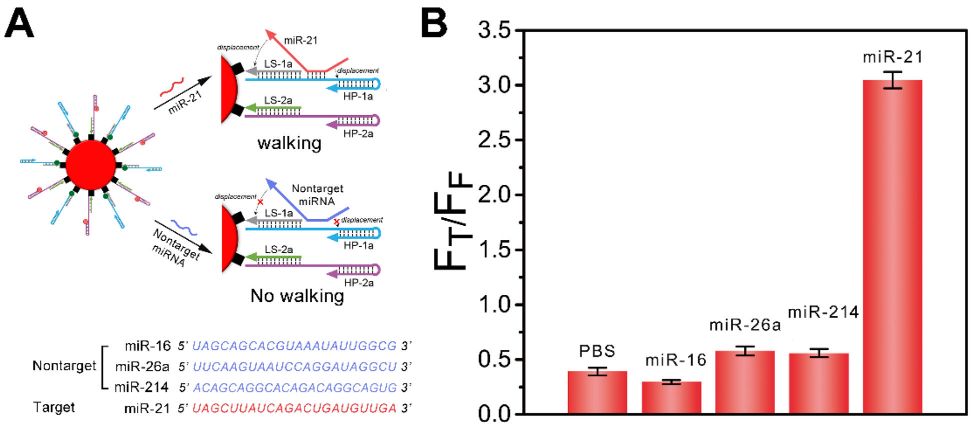

Figure S4 (A) Scheme showing the nanomachine treated by target miR-21 or nontarget miRNAs (miR-16, miR-26a, and miR-214). (B) Fluorescence intensity ratio (F_T/F_F) of the nanomachine in the presence of PBS, miR-16, miR-26a, miR-214, and miR-21.

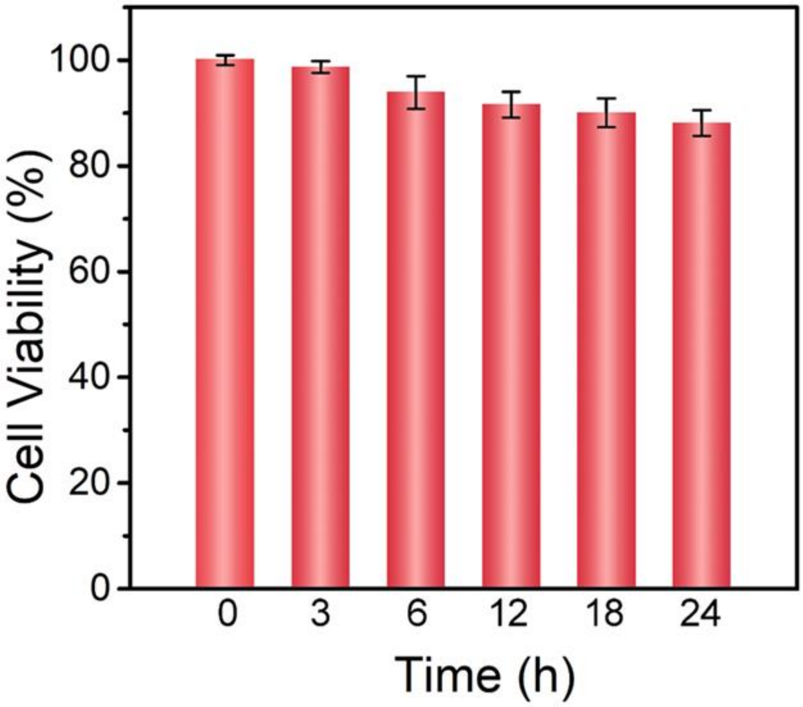

Figure S5 Cell viability determined by MTT cytotoxicity assays. MCF-7 cells were incubated with nanomachine (6 nM) for 0, 3, 6, 12, 18 and 24 h.

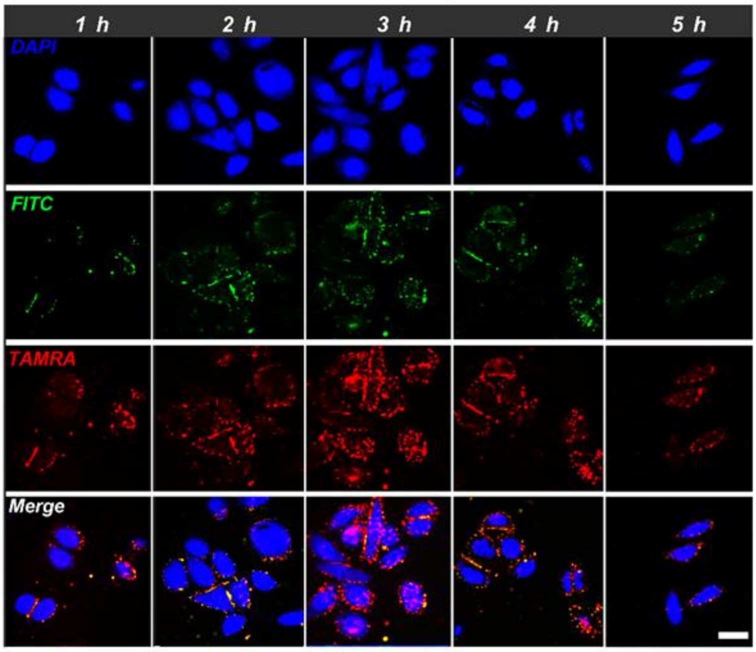

Figure S6 Confocal images of MCF-7 cells incubated with the nanomachine (6 nM) at different time points. Scale bar: 20 μm.

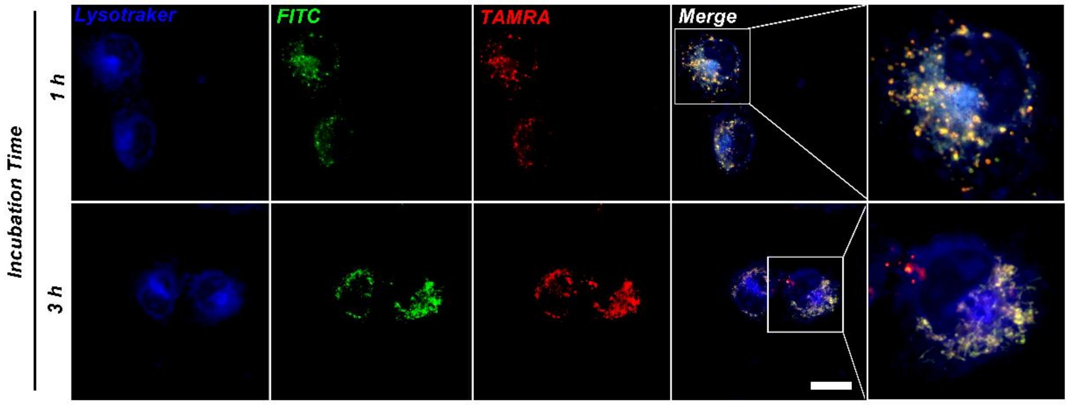

Figure S7 Confocal images of MCF-7 cells treated with LysoTracker blue and nanomachine for 1 h and 3 h, respectively. Scale bar: 20 μm.

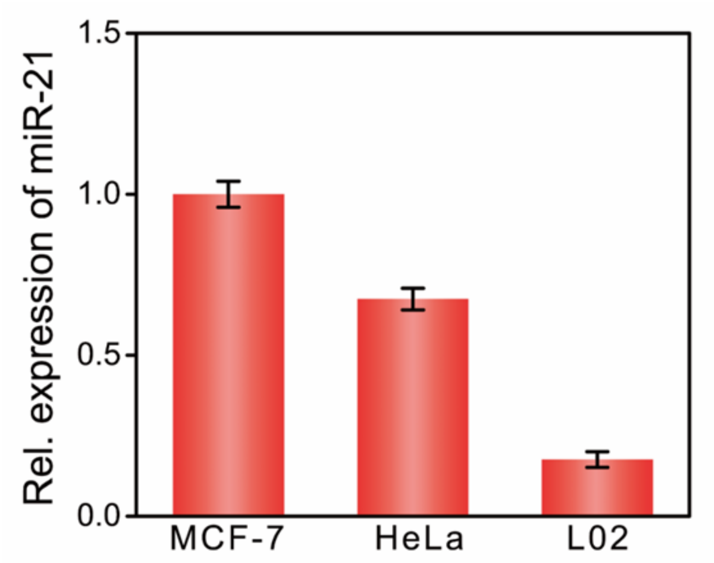

Figure S8 The relative expression levels of miR-21 in MCF-7, HeLa and L02 by qRT-PCR.

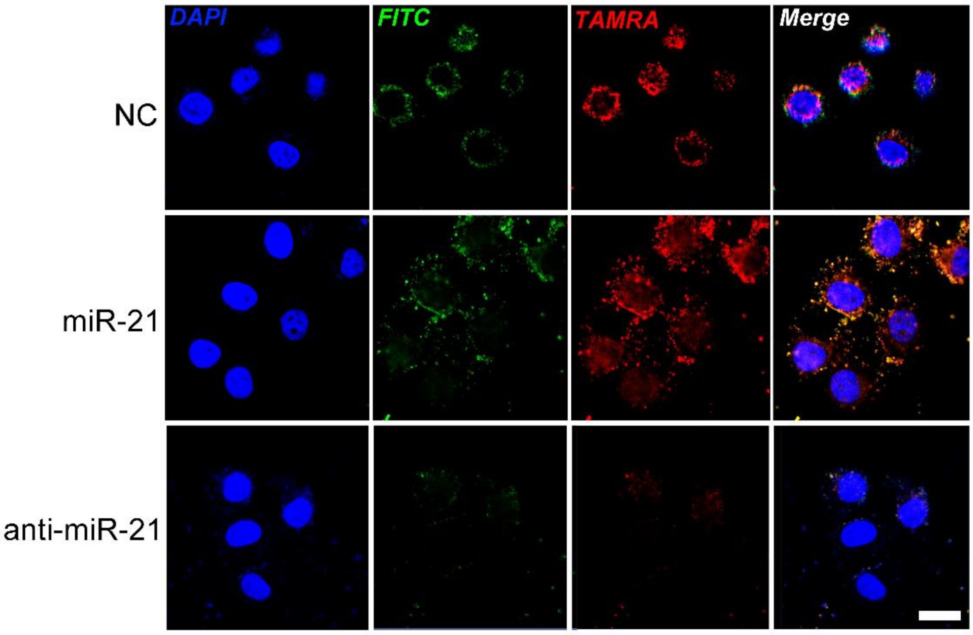

Figure S9 Confocal images of miR-21 in HeLa cells using the DNA walking nanomachine after transfecting the cells with NC (negative control), miR-21 mimic or inhibitor. Scale bar: 20 μm.

Table S1. Sequences of oligonucleotides.

Name	Sequence (5′ to 3′)
LS-1(4 nt)	CAGACTGATGTTGATTT-SH
LS-1(6 nt)	AGACTGATGTTGATTT-SH
LS-1(8 nt)	GACTGATGTTGATTT-SH
LS-2	CTTTGGGGTAGCTTTTT-SH
HP-1(4 nt)	TCAACATCAGTCTGATAAGCTACCCCTTTGGGGTAGC-FITC
HP-1 (6 nt)	TCAACATCAGTCTGATAAGCTACCCCTTTGGGGTAG-FITC
HP-1 (8 nt)	TCAACATCAGTCTGATAAGCTACCCCTTTGGGGTA-FITC
HP-2	TAMRA-GCTACCCCAAAGGGGTAGCTTATCAGACTGATAGTCTGATAA
Molecular beacon	FITC-TCAACATCAGTCTGATAAGCTAAAAAAAAAAGATGTTGA-BHQ1
miR-21	UAGCUUAUCAGACUGAUGUUGA
miR-21 mimic	UAGCUUAUCAGACUGAUGUUGA
miR-21 inhibitor	TCA ACATCAGTCTGATAAGCTA
miR-21 forward	GTGCAGGGTCCGAGGT
miR-21 reverse	GCCGCTAGCTTATCAGACTGATGT
U6 forward	CTCGCTTCGGCAGCACA
U6 reverse	AACGCTTCACGAATTTGCGT
miR-16	UAGCAGCACGUAAAUAUUGGCG
miR-26a	UUCAAGUAAUCCAGGAUAGGCU
miR-214	ACAGCAGGCACAGACAGGCAGU

Table S2. Comparison of the assay performance of the nanomachine with previously reported methods for intracellular miRNA detection.

	Method	LOD	Readout	Ref.
1	Au Nanoflare Probe	0.68 nM	Fluorescence	1
2	Isothermal Circular Strand Displacement Polymerization (B-ICSDP)	129.4 pM	Fluorescence	2
3	Hairpin-Fuelled Catalytic Nanobeacons	67 pM	Fluorescence	3
4	Single-Layer Perfluorinated Tungsten Diselenide Nanoplatform	0.75 nM	Fluorescence	4
5	DNAzyme Amplification Strategy	44 pM	Fluorescence	5
6	HCR System	680 pM	Fluorescence	6
7	DNA Walking Nanomachine	26 pM	Fluorescence	This work

References

1. Zhai LY, Li MX, Pan WL, Chen Y, Li MM, Pang JX, et al. In Situ Detection of Plasma Exosomal MicroRNA-1246 for Breast Cancer Diagnostics by a Au Nanoflare Probe. ACS Appl Mater Inter. 2018; 10(46): 39478-39486.
2. Yang Z, Zhang S, Zhao H, Niu H, Wu ZS, Chang HT. Branched DNA Junction-Enhanced Isothermal Circular Strand Displacement Polymerization for Intracellular Imaging of MicroRNAs. Anal Chem. 2018; 90: 13891-13899.
3. Wang J, Huang J, Quan K, Li J, Wu Y, Wei Q, et al. Hairpin-Fuelled Catalytic Nanobeacons for Amplified MicroRNA Imaging in Live Cells. Chem Commun. 2018; 54(73): 10336-10339.
4. Song Y, Yan X, Ostermeyer G, Li S, Qu L, Du D, et al. Direct Cytosolic MicroRNA Detection Using Single-Layer Perfluorinated Tungsten Diselenide Nanoplatform. Anal Chem. 2018; 90(17): 10369-10376.
5. Li P, Wei M, Zhang F, Su J, Wei W, Zhang Y, et al. Novel Fluorescence Switch for MicroRNA Imaging in Living Cells. ACS Appl Mater Inter. 2018; 10(50): 43405-43410.
6. Yang F, Cheng Y, Cao Y, Dong H, Lu H, Zhang K, et al. Sensitively Distinguishing Intracellular Precursor and Mature MicroRNA Abundance. Chem Sci. 2018; 10(6): 1709-1715.
